# Brief Communication: Histological Assessment of Nonhuman Primate Brown Adipose Tissue Highlights the Importance of Sympathetic Innervation

**DOI:** 10.1155/2023/5651084

**Published:** 2023-01-19

**Authors:** Abigail G. Williams, Masha Long, Kylie Kavanagh

**Affiliations:** Department of Pathology, Wake Forest University School of Medicine, Winston-Salem, NC, USA

## Abstract

**Objective:**

The objective of this study was to functionally analyze the correlation of key histological features in brown adipose tissue (BAT) with clinical metabolic traits in nonhuman primates.

**Methods:**

Axillary adipose tissue biopsies were collected from a metabolically diverse nonhuman primate cohort with clinical metabolism-related data. Expression of tyrosine hydroxylase (TH), uncoupling protein 1 (UCP1), cluster of differentiation 31 (CD31), cytochrome c oxidase subunit 4 (COX IV), beta-3 adrenergic receptor (*β*3-AR), and adipose cell size were quantified by immunohistochemical analysis. Computed tomography scans were performed to assess body composition.

**Results:**

Tyrosine hydroxylase was negatively correlated with whole body fat mass as a percentage of body weight (*p* = 0.004) and was positively correlated with the density of UCP1 (*p* = 0.02), COX IV (*p* = 0.006), CD31 (*p* = 0.007), and cell density (*p* = 0.02) of the BAT samples. Beta-3 adrenergic receptor abundance had a weak positive correlation with COX IV (*p* = 0.04) in BAT but did not significantly correlate to UCP1 or TH expression in BAT.

**Conclusions:**

Our findings highlight that there is a disparity in innervation provided to BAT based on body composition, as seen with the negative association between TH, a marker for innervation, and adiposity. These findings also support the importance of innervation in the functionality of BAT, as TH abundance not only supports leaner body composition but is also positively correlated with known structural elements in BAT (UCP1, COX IV, CD31, and cell density). Based on our observations, *β*3-AR abundance does not strongly drive these structural elements or TH, all of which are known to be important in the function of brown adipose tissue. In effect, while the role of other receptors, such as *β*2-AR, should be reviewed in BAT function, these results support the development of safe sympathetic nervous system stimulants to activate brown adipose tissue for obesity treatment.

## 1. Introduction

Obesity is associated with health problems such as high blood pressure, type 2 diabetes, heart disease, and other metabolic illnesses. With roughly 42.4% of the United States' adult populace diagnosed with obesity in 2018, new treatment methodologies are essential [1]. An emerging target tissue for treatment of obesity and other metabolic diseases is brown adipose tissue (BAT). BAT is a metabolically active tissue, in which uncoupling protein 1 (UCP1) uncouples mitochondrial oxidation from ATP synthesis and dissipates energy in the form of heat during the natural sympathetic response to cold exposure [2]. Fifty grams of activated brown fat can account for roughly 20% of whole-body energy expenditure [3]. There have been attempts to stimulate BAT pharmacologically with the use of sympathomimetics, but adverse cardiologic effects have limited progression of possible agents to clinical use [4].

There has been recent imaging success towards understanding activated BAT *in vivo*, but there is a lack of data concerning the relationship of multiple in situ BAT tissue features, which is vital to identify and understand BAT functionality before it can be utilized in a clinical setting [5–7]. The goal of this study was to assess key BAT histological features relating to mitochondria and its uncoupling, as well as innervation and vascularization from in situ tissues collected from adult nonhuman primates (NHPs) encompassing a range of adiposity and health statuses. NHPs allow for a more relevant interpretation than other animal models due to the close physiological relationship between NHPs and people. With the failure of many pharmaceutical candidates to translate to effective human therapies, particularly for cardiometabolic diseases, understanding BAT biology in NHPs can aid drug development and research directions [8].

We found that axillary BAT in adult NHPs histologically resembles that in humans and functionally compares to previous rodent studies that indicate BAT functionality impacts obesity characteristics [9]. Here, we show that tyrosine hydroxylase, an innervation biomarker in BAT that is a fundamental component of norepinephrine synthesis, has the highest relationship with body composition and that *β*3-AR abundance does not drive brown fat functionality. These findings provide translational support for pharmacological targeting of the sympathetic nervous system in the treatment of obesity but suggest that other ARs be reviewed for their relationships with brown fat functionality.

## 2. Methods

### 2.1. Nonhuman Primates

The NHP cohort (*n* = 26) was comprised of 11 female African green monkeys (*Chlorocebus aethiops sabaeus*) from the Vervet Research Colony at the Wake Forest University School of Medicine (WFUSM) and 15 male rhesus macaques (*Macaca mulatta*) [10], also housed at the WFUSM. The cohort was selected to be diverse in age and metabolic health; 7 individuals presented with type 2 diabetes, 4 presented with obesity, and 8 presented with hypertension (Tables S1 and S2). All health phenotypes presented spontaneously, and animal procedures were approved by the Wake Forest University Institutional Animal Care and Use Committee.

### 2.2. Adipose Biopsies and Immunohistochemistry

Tissues collected from each individual included axillary BAT and paraumbilical subcutaneous white adipose tissue (WAT). Tissue was fixed in 10% neutral buffered formalin and embedded in paraffin wax prior to 4-5 *μ*m thick tissue sections being mounted on charged slides and stained with cluster of differentiation 31 ((CD31) Cat No. CM131C; Biocare Medical, Pacheco, CA**)**, cytochrome c oxidase subunit 4 ((COX IV) Cat No. MA5-15078; Thermo Fisher Scientific, Waltham, MA), tyrosine hydroxylase ((TH) Cat No. MAB318; MilliporeSigma, Burlington, MA), uncoupling protein 1 ((UCP1) Cat No. PA1-24894; Thermo Fisher Scientific, Waltham, MA), and beta-3 adrenergic receptor ((*β*3-AR) Cat No. Ab140713; Abcam, Cambridge, UK) antibodies. WAT samples were stained for CD31, while BAT samples were stained for CD31, COX IV, TH, UCP1, and *β*3-AR. Control samples were reviewed for staining verification (Figure S1). Slides were digitally scanned, and image analysis was completed by an operator blinded to health status using Visiopharm software (Hørsholm, Denmark). Analyses included the whole tissue sample and a visually representative region of interest, with results expressed as the percent positive stain by the tissue area. The UCP1 measures were expressed as *z*-scores, as data were generated from separate batches of immunostaining. The *β*3-AR samples were scored (1: low abundance of *β*3-AR stain to 4: high abundance of *β*3-AR stain) based on the overall coverage of an antibody by 5 individuals blinded to health status. An average cell size for each subject was calculated based on a representative region of interest within the sample, and nucleus density was calculated as the total nucleus area divided by the total tissue area. To ensure that BAT depots were comparable, axillary BAT samples were compared to supraclavicular BAT samples in the rhesus macaques, and there were no significant regional differences in brownness, measured by the abundance of the UCP1 antibody present (Table S3).

### 2.3. Body Composition

Computed tomography scans were performed within 3 months of tissue collections (Siemens SOMATOM Definition Flash, Munich, Germany) to establish total body composition characteristics including total body fat and lean mass percentages in relation to body weight at the time of scans (Table S1, Figure S2). The scans were reconstructed as DICOM images using AquariusNet Thin Client (TeraRecon, Durham, NC) and then analyzed as previously described [10] using Materialise's interactive medical image control system ((Mimics) Materialise, Leuven, Belgium) (Table S1, Figure S2).

### 2.4. Data Analysis

Statistical analysis was performed using Statistica v13 (StatSoft, Tulsa, OK). Body weight, fasting blood glucose, and diastolic blood pressure were analyzed as *z*-scores due to species or sex-specific differences. All continuous variables were assessed for normality and were log transformed if needed to achieve a normal distribution. Significant correlations were assessed by Pearson's correlation coefficients, controlled for age, and significance was assumed when *p* ≤ 0.05; trends were indicated when *p* ≤ 0.10 for interval scale data. Significant correlations were assessed by Spearman Rank correlation coefficients, and significance was assumed when *p* ≤ 0.05; trends were indicated when *p* ≤ 0.10 for ordinal data.

## 3. Results

We characterized the metabolic diversity of our NHP cohort by using metrics comparable to human metabolic outcomes (Table S1). We measured age, body weight, systolic and diastolic blood pressures, fasting blood glucose, hemoglobin A1c, triglyceride concentration, high-density lipoprotein cholesterol (HDLC) concentration, adiposity represented by fat mass as a percentage of body weight, and cell density (Table S1). We found that our cohort was diverse and presented healthy subjects, as well as those with hypertension, hyperglycemia, and obesity across both species (Table S2).

**Figure 1:**
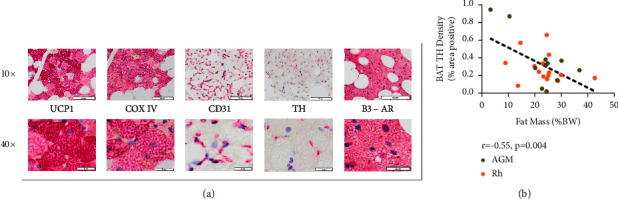
Immunohistochemical analysis of brown adipose tissue. Panel (a) depicts the histological features, UCP1, COX IV, CD31, TH, and *β*3-AR, as expressed in brown adipose tissue from a healthy African green vervet monkey. The immunostaining from this subject was chosen for presentation here, as this subject has the highest antibody abundance across the most histological features measured. A healthy high abundance sample was chosen to provide a clear depiction of immunostaining itself. The top row of the panel displays samples of adipose tissue with indicated antibodies at 10x magnification, and the bottom row displays a portion of the same region of interest at 40x magnification. The graphic on panel (b) illustrates the significant correlation between brown adipose tissue tyrosine hydroxylase (TH) density and fat mass expressed as a percentage of body weight (*p* value = 0.004, *r* = −0.55, *n* = 26).

**Figure 2:**
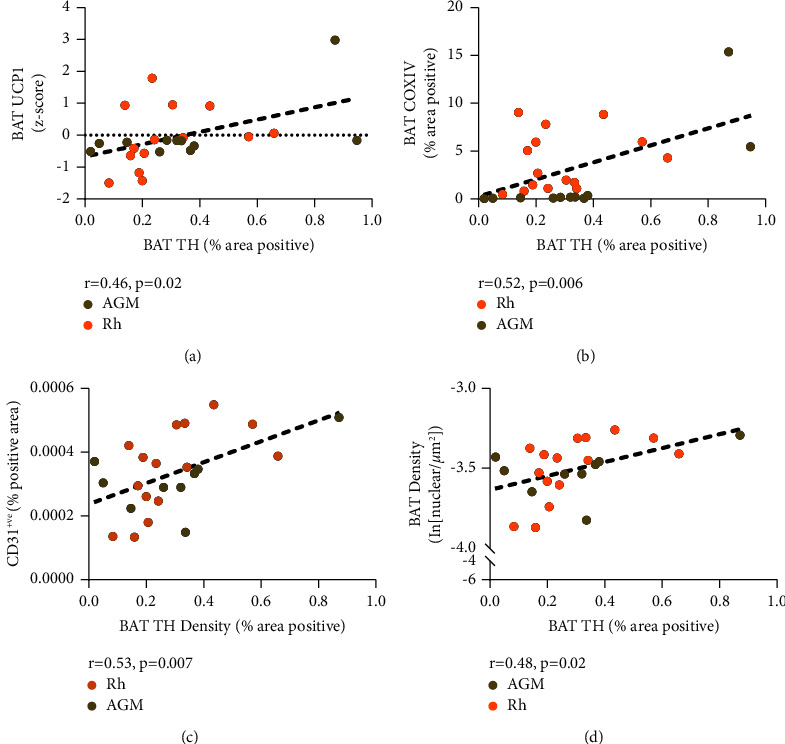
Significant histological features and BAT density correlates of tyrosine hydroxylase density. Panel (a) depicts the positive correlation between uncoupling protein 1 (UCP1) and tyrosine hydroxylase (TH) (*p* value = 0.02, *r* = 0.46, *n* = 26). Panel (b) depicts the positive correlation between cytochrome c oxidase subunit 4 (COX IV) and TH (*p* value = 0.006, *r* = 0.52, *n* = 26). Panel (c) depicts the positive correlation between cluster of differentiation 31 (CD31) and TH (*p* value = 0.007, *r* = 0.52, *n* = 24). Panel (d) depicts the positive correlation between brown adipose tissue (BAT) cell density and TH (*p* = 0.02, *r* = 0.48, *n* = 24).

**Figure 3:**
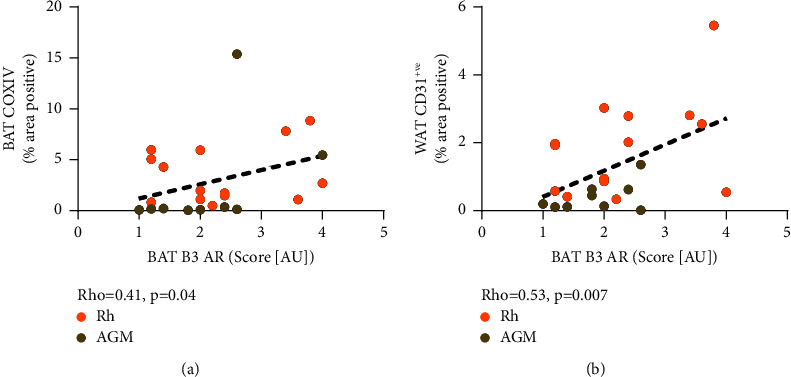
Significant correlations with the beta 3 adrenergic receptor. Panel (a) depicts the positive relationship between abundance of beta-3 adrenergic receptor (*β*3-AR) and cytochrome c oxidase subunit 4 (COX IV) (*p* value = 0.04, Rho = 0.41, *n* = 25) in brown adipose tissue. Panel (b) depicts the positive relationship between brown adipose tissue beta-3 adrenergic receptor (*β*3-AR) abundance and white adipose tissue cluster differentiation 31 (CD31) (*p* value = 0.007, Rho = 0.53, *n* = 25).

We completed the immunohistochemical analysis of adipose tissue stained with UCP1, COX IV, CD31, TH, and *β*3-AR antibodies to measure brown fat abundance (UCP1), mitochondrial abundance (UCP1 and COX IV), perfusion (CD31), sympathetic innervation provided to tissue (TH), and adrenergic receptor abundance (*β*3-AR) (Figure 1(a)). We found that BAT TH was the only histological feature that had a significant correlation with a clinical metabolic measure, as it was negatively correlated with adiposity (*p* value = 0.004) (Figure 1(b)). We found that BAT TH density was in turn positively correlated with other key features of BAT, including UCP1 (*p* value = 0.02), COX IV (*p* value = 0.006), CD31 (*p* value = 0.007), and cell density (*p* value = 0.02) (Figure 2). The *β*3-AR abundance in BAT had a strong positive relationship with CD31 coverage in WAT (*p* value = 0.007) and a weak yet significant positive relationship with COX IV in BAT (*p* value = 0.04) (Figure 3).

## 4. Discussion

There is a gap in knowledge regarding the association of key BAT histological features, UCP1, COX IV, TH, CD31, and *β*3-AR, and expressed clinical phenotypes. To date, there has not been a study that analyzes innervation, endothelial, and mitochondrial markers within BAT explants. Innervation is highly significant in BAT functionality as it is responsible for catalyzing the reaction of events necessary for UCP1 activation, and thus the energy expensive process of UCP1-dependent thermogenesis itself. This occurs through the stimulation and release of norepinephrine by the sympathetic nervous system in response to cold exposure, which binds to beta-adrenergic receptors (*β*-ARs), leading to the production of cyclic adenosine monophosphate and activation of UCP1 [11].

There have been attempts to increase BAT activation with sympathomimetics, as it is a tissue capable of extensive energy expenditure [3], in order to treat obesity and diabetes by mimicking the effect of norepinephrine on *β*3-AR. However, side effects including increased heart rate and elevated blood pressure have hampered drug development to date [4]. There has also been recent debate as to the prevalence of *β*2-AR over *β*3-AR in human brown fat, which has been hypothesized why a promising *β*3-AR agonist, mirabegron, has displayed conflicting results in preclinical testing [12–14].

There are several reasons why BAT innervation may vary between individuals. It is known that age can be related to impairment of the sympathetic nervous system, as well as to changes in endocrine signals and inflammation [15, 16]. Exercise also plays an important role in sympathetic tone, and previous research suggests that exercise increased sympathetic tone in BAT [17].

The findings of this study were reproducible across two NHP species that were different in size, sex, and age; however, each NHP species displays the same range of metabolic health from lean and healthy to obese and type 2 diabetic (Tables S1 and S2). This demonstrates the reproducibility and generalizability of the relationship of BAT TH, and other histological features, with obesity and related metabolic disorders. Our findings concerning tyrosine hydroxylase provide evidence that supports the use of sympathomimetics to activate BAT in the treatment of diabetes, obesity, and other cardiometabolic disorders, as they illustrate the importance of innervation in the functionality of brown adipose tissue. Furthermore, our results suggest that the abundance of *β*3-AR does not drive brown adipose tissue functionality, indicating that other *β*-ARs should be reviewed in relation to BAT functionality and considered for sympathomimetic drug development. These characterizations of nonhuman primate adipose also highlight the need for further study into the reasons for variation in BAT innervation.

## Data Availability

There are immunohistochemical and imaging data, as well as historical clinical data on the subjects, available upon request.

## Additional Points

*Study Importance Questions.* (1) What is already known about this subject? (a) Brown adipose tissue is currently targeted as a possible treatment avenue for obesity. One possible treatment method is through the use of sympathetic nervous system stimulants. (2) What are the new findings in our manuscript? (a) We survey key histological features of primate brown adipose tissue to collate the relative importance of innervation, perfusion, cell size/density, and mitochondria-related markers, which, to date, have not been evaluated in relation to obesity. (b) In our metabolically diverse and translationally relevant nonhuman primate cohort, tyrosine hydroxylase density, a marker for innervation, is significantly negatively correlated with body fat percentage. (c) Beta-3 adrenergic receptor abundance does not correlate with brown adipose tissue innervation as measured by TH nor to most metabolic histological markers. (3) How might our results change the direction of research or the focus of clinical practice? (a) Our findings highlight that high abundance of innervation of brown adipose tissue correlates with lower fat mass and supports the use of sympathetic nervous system stimulants to activate brown adipose tissue to treat obesity. (b) These results also call for more investigation into processes that cause disparity in innervation provided to and received by brown adipose tissue.

## Supplementary Materials

Supplementary Materials Table S1: descriptive table of clinical variables measured within the cohorts. This table includes the mean and standard error mean values for age (years), bodyweight (kg), systolic blood pressure (mmHg), diastolic blood pressure (mmHg), fasting glucose (mg/dL), A1c (%), TG (mg/dL), HDLC (mg/dL), adiposity (fat mass as % body weight), cell density (nuclei area/total area), whole body fat tissue (% of body weight), and whole body lean tissue (% of body weight). The values are for each species as well as the entire cohort. *p* values are derived from one-way ANOVA and indicative of any species-related differences (significance: *p* value ≤0.05). Table S2: prevalence of clinically diagnosed metabolic disorders within the nonhuman primates. This table displays prevalence of hypertension, hyperglycemia, and obesity within each species cohort. The cohorts were matched based on the proportions of unhealthy individuals in which brown adipose characteristics were assessed. Figure S1: immunohistochemical staining control samples. This figure displays a panel at 4x magnification of control samples of each of the 5 main antibodies assessed in this study (UCP1, COX IV, CD31, TH, and *β*3-AR). Table S3: comparison of UCP1 content between supraclavicular and axillary BAT depots. This table displays the mean and standard deviation of the UCP1 percent area across supraclavicular and axillary BAT depots of 4 rhesus macaques. A paired *t*-test determined no significant difference between the UCP1 percent area in either depot. Figure S2: body composition calculated from computed tomography scans. This figure depicts fat and lean tissue thresholds for animals with the lowest and highest body fat percentage within the rhesus macaque cohort (similar to as seen in the vervets). We were able to derive the percentage of body weight attributed to fat and lean tissue and characterize adiposity from these thresholds.
