# Supplementary material for: Brief Communication: Histological Assessment of Nonhuman Primate Brown Adipose Tissue Highlights the Importance of Sympathetic Innervation
Source: J Obes. 2023 Jan 19;2023:5651084. doi: 10.1155/2023/5651084 (PMC9879676; doi:10.1155/2023/5651084)
Supplement: Supplementary Materials — Table S1: descriptive table of clinical variables measured within the cohorts. This table includes the mean and standard error mean values for age (years), bodyweight (kg), systolic blood pressure (mmHg), diastolic blood pressure (mmHg), fasting glucose (mg/dL), A1c (%), TG (mg/dL), HDLC (mg/dL), adiposity (fat mass as % body weight), cell density (nuclei area/total area), whole body fat tissue (% of body weight), and whole body lean tissue (% of body weight). The values are for each species as well as the entire cohort. p values are derived from one-way ANOVA and indicative of any species-related differences (significance: p value ≤0.05). Table S2: prevalence of clinically diagnosed metabolic disorders within the nonhuman primates. This table displays prevalence of hypertension, hyperglycemia, and obesity within each species cohort. The cohorts were matched based on the proportions of unhealthy individuals in which brown adipose characteristics were assessed. Figure S1: immunohistochemical staining control samples. This figure displays a panel at 4x magnification of control samples of each of the 5 main antibodies assessed in this study (UCP1, COX IV, CD31, TH, and β3-AR). Table S3: comparison of UCP1 content between supraclavicular and axillary BAT depots. This table displays the mean and standard deviation of the UCP1 percent area across supraclavicular and axillary BAT depots of 4 rhesus macaques. A paired t-test determined no significant difference between the UCP1 percent area in either depot. Figure S2: body composition calculated from computed tomography scans. This figure depicts fat and lean tissue thresholds for animals with the lowest and highest body fat percentage within the rhesus macaque cohort (similar to as seen in the vervets). We were able to derive the percentage of body weight attributed to fat and lean tissue and characterize adiposity from these thresholds. [file 5651084.f1.docx]

**Supplemental Data File**

**Brief Communication: Histological Assessment of Nonhuman Primate Brown Adipose Tissue Highlights the Importance of Sympathetic Innervation**

AG Williams^1,2^, M Long^1,3^, K Kavanagh^1,4^

^1^ Department of Pathology, Wake Forest University School of Medicine, Winston-Salem, NC, USA

^2^ abwillia@wakehealth.edu

^3^ mblock@wakehealth.edu

^4^ kkavanag@wakehealth.edu

| **Mean (± SEM)** | **Vervet (n = 11)** | **Rhesus (n = 13-15)** | **Cohort (n = 26)** | **p - value** |
| --- | --- | --- | --- | --- |
| **Sex** | Female | Male | - | - |
| **Age (years)** | 16.91 ( ± 1.05) | 9.20 ( ± 0.11) | 12.46 ( ± 0.88) | 9.18*10^-9^ |
| **Bodyweight (kg)** | 6.12 ( ± 0.40) | 15.76 ( ± 0.85) | 11.68 ( ± 1.08) | 2.63*10^-9^ |
| **Systolic BP (mmHg)** | 122.58 ( ± 7.72) | 117.87 ( ± 3.92) | 119.86 ( ± 3.91) | 0.56 |
| **Diastolic BP (mmHg)** | 70.77 ( ± 2.92) | 58.67 ( ± 3.65) | 63.79 ( ± 2.68) | 0.02 |
| **Fasting Glucose (mg/dL)** | 194.64 ( ±46.93) | 95.13 ( ± 15.60) | 137.23 ( ± 23.41) | 0.03 |
| **A1c (%)** | 5.71 ( ± 0.76) | 6.18 ( ± 0.75) | 5.96 ( ± 0.53) | 0.67 |
| **TG (mg/dL)** | 86.36 ( ± 11.05) | 160.00 ( ± 86.91) | 128.85 ( ± 50.14) | 0.48 |
| **HDLC (mg/dL)** | 108.75 ( ± 13.00) | 95.69 ( ± 4.32) | 101.68 ( ± 6.79) | 0.35 |
| **Adiposity (Fat Mass as % BW)** | 22.68 ( ± 2.76) | 23.40 ( ± 2.02) | 23.10 ( ± 1.62) | 0.83 |
| **Cell Density (Nuclei Area / Total Area)** | 9.73*10^-3^ ( ± 0.01) | 12.00*10^-3^ ( ± 0.01) | 11.00*10^-3^ ( ± 0.01) | 0.31 |
| **Whole Body Fat Tissue (% of Body Weight)** | 22.68 (± 2.76) | 23.40 (± 2.02) | 23.10 (± 1.61) | 0.83 |
| **Whole Body Lean Tissue (% of Body Weight)** | 53.06 (± 2.07) | 53.67 (± 1.56) | 53.41 (± 1.23) | 0.81 |

**Table S1: Descriptive table of clinical variables measured within the cohorts.** Indicators of declining metabolic health were: fasting blood glucose ≥ 100 mg/dL, glycosylated hemoglobin [A1c] ≥ 6, plasma triglycerides [TG] ≥ 150, high density lipoprotein cholesterol [HDLC] ≥ 50, systolic blood pressure [BP] ≥ 120, and diastolic BP ≥ 80. Nonhuman primate availability led to more diabetics in the vervet group than rhesus group (see Table S2). P – values were derived from one-way ANOVA and indicative of any species related differences (significance: p-value ≤ 0.05).

|  | **Vervet**  **(# with condition)** | **Rhesus**  **(# with condition)** | **χ^2^ stat (p-value)** |
| --- | --- | --- | --- |
| **Hypertension** | 2 | 6 | 1.42 (0.23) |
| **Hyperglycemia** | 4 | 3 | 0.86 (0.35) |
| **Obesity** | 2 | 2 | 0.11 (0.74) |

**Table S2:** **Prevalence of clinically diagnosed metabolic disorders within the nonhuman primates.** Hypertension was classified by three consistent measures of high systolic and/or diastolic blood pressure as described in Table S1. Diabetes was classified by meeting both criteria for elevated fasting blood glucose and A1c% for three consecutive measures as described in Table S1. Obesity was defined as a body fat percentage ≥ 30% from image analyses of computed tomography scans. Cohorts were matched on the proportions of unhealthy individuals in which brown adipose characteristics were assessed.

**
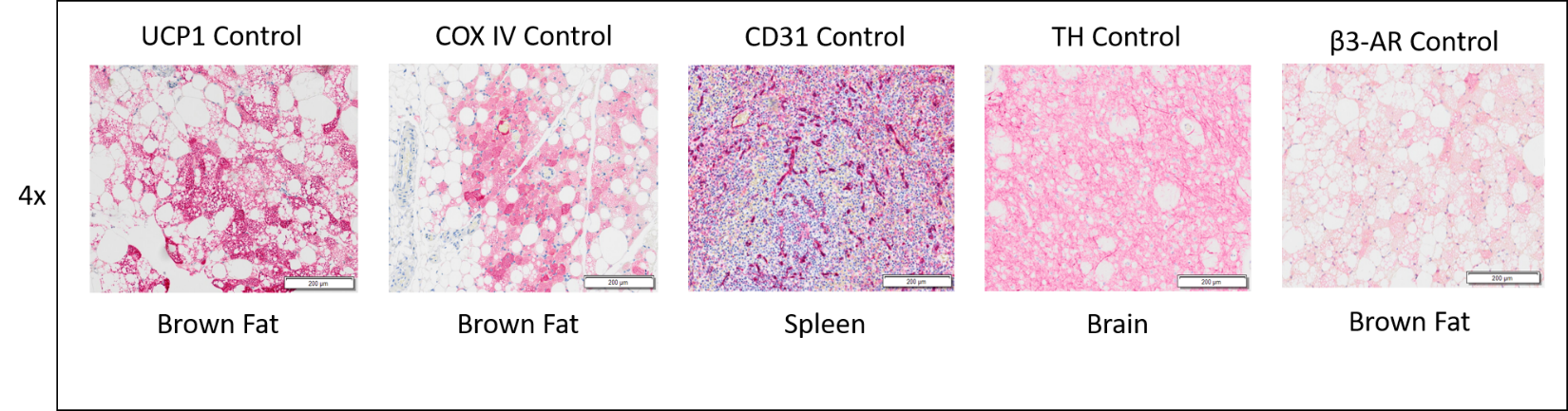
**

**Figure S1: Immunohistochemical Staining Control Samples.** All control samples were collected from African Green monkeys (*Chlorocebus aethiops sabaeus;* vervets). The uncoupling protein 1 (UCP1) and cytochrome c oxidase subunit 4 (COX IV) control samples were from brown adipose tissue. The cluster of differentiation 31 (CD31) control is a staining of spleen tissue, the tyrosine hydroxylase (TH) control is a staining of brain tissue, and the beta 3 adrenergic receptor (β3-AR) is a staining of brown adipose tissue.

|  | **Mean (Std. Deviation)** | **N** | **P-Value** |
| --- | --- | --- | --- |
| **Supraclavicular % Area UCP1** | 21.83 (± 18.48) | 4 | 0.67 |
| **Axillary % Area UCP1** | 28.97 (± 18.05) | 4 |  |

**Table S3**: **Comparison of UCP1 Content between Supraclavicular and Axillary BAT Depots.** Both supraclavicular and axillary BAT samples were derived at the same time from the same 4 rhesus macaques. A paired t-test determined no significant difference between the uncoupling protein 1 (UCP1) percent area in either depot (p-value = 0.67, N = 4, test stat = -0.46, df = 3).


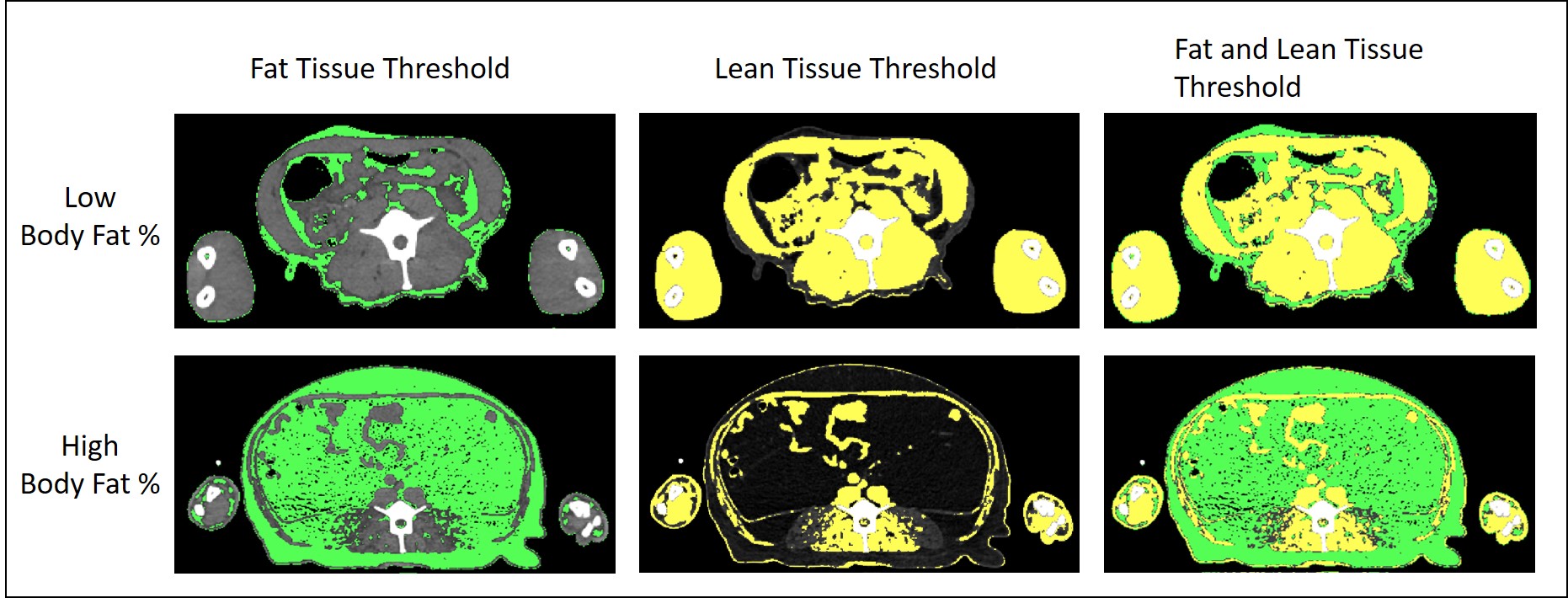


**Figure S2: Body Composition Calculated from Computed Tomography Scans.** Depicted are fat (-140 to – 40 HU) and lean (-5 to 135 HU) tissue thresholds for the animals with the lowest and highest body fat percentage within the rhesus macaque cohort (similar to as seen in the vervets). From these thresholds, we were able to derive the percentage of body weight attributed to fat tissue and lean tissue and characterize individuals as obese (>30% of body weight as adipose).
